# Redox Signaling and Advanced Glycation Endproducts (AGEs) in Diet-Related Diseases

**DOI:** 10.3390/antiox9020142

**Published:** 2020-02-06

**Authors:** Vanesa Cepas, Massimo Collino, Juan C. Mayo, Rosa M. Sainz

**Affiliations:** 1 Departamento de Morfologia y Biologia Celular, Redox Biology Group, Universidad de Oviedo, 33403 Oviedo, Spain; cepasvanesa@uniovi.es; 2 Instituto Universitario de Oncologia del Principado de Asturias (IUOPA), Universidad de Oviedo, 33403 Oviedo, Spain; 3 Dipartimento di Scienza e Tecnologia del Farmaco, Università degli Studi di Torino, 10125 Torino, Italy; Massimo.collino@unito.it

**Keywords:** advanced glycation products, RAGE, diet-related diseases, antioxidants

## Abstract

Diets are currently characterized by elevated sugar intake, mainly due to the increased consumption of processed sweetened foods and drinks during the last 40 years. Diet is the main source of advanced glycation endproducts (AGEs). These are toxic compounds formed during the Maillard reaction, which takes place both in vivo, in tissues and fluids under physiological conditions, favored by sugar intake, and ex vivo during food preparation such as baking, cooking, frying or storage. Protein glycation occurs slowly and continuously through life, driving AGE accumulation in tissues during aging. For this reason, AGEs have been proposed as a risk factor in the pathogenesis of diet-related diseases such as diabetes, insulin resistance, cardiovascular diseases, kidney injury, and age-related and neurodegenerative diseases. AGEs are associated with an increase in oxidative stress since they mediate the production of reactive oxygen species (ROS), increasing the intracellular levels of hydrogen peroxide (H_2_O_2_), superoxide (O_2_^−^), and nitric oxide (NO). The interaction of AGEs with the receptor for AGEs (RAGE) enhances oxidative stress through ROS production by NADPH oxidases inside the mitochondria. This affects mitochondrial function and ultimately influences cell metabolism under various pathological conditions. This short review will summarize all evidence that relates AGEs and ROS production, their relationship with diet-related diseases, as well as the latest research about the use of natural compounds with antioxidant properties to prevent the harmful effects of AGEs on health.

## 1. Introduction: General Aspects of Advanced Glycation Endproducts

**Figure 1:**
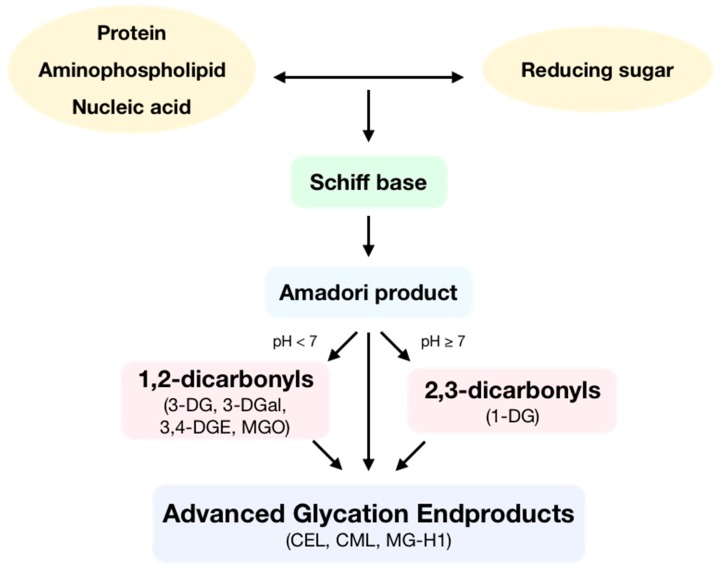
Formation of advanced glycation endproducts (AGEs). During the Maillard reaction, the free amino group of a protein, aminophospholipid, or nucleic acid reacts with the carbonyl group of a reducing sugar, producing a Schiff base. This molecule, following some rearrangement, is spontaneously transformed into an Amadori product. Depending on pH, these products form 1,2-dicarbonyls as 3-deoxyglucosone (3-DG), 3-deoxygalactosone (3-DGal), or methylglyoxal (MGO) or form 2,3-dicarbonyls such as 1-deoxyglucosone (1-DG). Then, dicarbonyls following condensation produce AGEs such as Nε-(carboxyethyl)lysine (CEL), Nε-(carboxymethyl)lysine (CML) or methylglyoxal-derived hydroimidazolone 1 (MG-H1).

Advanced glycation endproducts (AGEs) are toxic compounds that are formed during the spontaneous reaction initiated by a nucleophilic addition between the free amino group of a protein, aminophospholipid or nucleic acid and the carbonyl group of a reducing sugar, called the Maillard reaction (Figure 1). In this reaction, a reversible Schiff base is formed, which is then spontaneously transformed at physiological pH and room temperature into an Amadori product after some rearrangement. Then, these Amadori products can react following two different routes depending on pH. At low pH values, enolization reactions take place to form 1,2-dicarbonyls, which can later dehydrate to yield furfural derivatives. Otherwise, at high pH values, enolization reactions occur, which produce 2,3-dicarbonyls that then dehydrate to yield reductones. Moreover, Amadori products can also form α-dicarbonyls through oxidative fission or retro-aldol fragmentation. Dicarbonyls are very reactive compounds. All the generated carbonyls can undergo condensation with primary amines to make melanoidins [1]. Then, AGEs can be formed by two different pathways: the irreversible rearrangement of Amadori products through both oxidative and non-oxidative pathways and through subsequent condensation reactions between dicarbonyls and the side-chain of lysine, cysteine, and arginine residues [2].

Protein glycation takes place in vivo in tissues and fluids under physiological conditions. It is a slow and continuous process that occurs throughout the lifespan, driving AGE accumulation in tissues during aging [3]. Nevertheless, protein glycation can also take place ex vivo, since this reaction occurs during food preparation such as baking, cooking, or frying as well as during storage [4]. High temperatures and long cooking times favor glycation reactions [5]. 

Diet is the major source of AGEs in vivo because AGEs are exogenously introduced with the diet and can also be produced endogenously from a diet with a high content of simple sugars, especially fructose [6]. This sugar is the most common in the human diet due to the high-fructose syrups usually added to processed foods and drinks as sweeteners. Furthermore, fructose is also 7.5-fold more reactive than glucose [7]. However, even if glucose is less reactive, it still plays an essential role in protein glycation since it can be transformed into fructose through the polyol pathway [8]. Furthermore, the accumulation of AGEs both in plasma and tissues has been reported in animal models of high fructose consumption, [9,10]. 

## 2. Advanced Glycation Endproducts Drive Cell Signaling and Inflammation

The action of AGEs is mediated via their receptors: AGE-R1/OST-48, AGE-R2/80K-H, AGE-R3/galectin-3, LOX-1 [11], and CD36 [12], implicated in the capture, removal, and degradation of AGEs, as well as the receptor for AGEs (RAGE); the latter is the most important and the most studied [13]. 

RAGE is a multiligand member of the immunoglobulin superfamily of type I cell surface molecules. It is a pattern recognition receptor, expressed in different cell types such as fibroblasts, keratinocytes, monocytes, macrophages, lymphocytes, endothelial, smooth muscle, and dendritic cells as well as neurons, glia, and chondrocytes [14]. It engages different ligands, not only AGEs, but also the amyloid β peptide, S100/calgranulin protein, HMGB1, and LPS, ultimately leading to an alteration in gene expression [15,16,17,18]. Recent studies have shown that Ne(carboxymethyl)lysine (CML) adducts of proteins, the most frequent type of AGEs found in vivo, interact with RAGE to activate signal transduction pathways [19,20], ultimately leading to the expression of proinflammatory genes [21]. Thus, it has been shown that treating cells with AGEs produces a rapid increase in both mRNA and protein levels of RAGE [22] which is suppressed after pretreatment with the anti-RAGE antibody [23].

**Figure 2:**
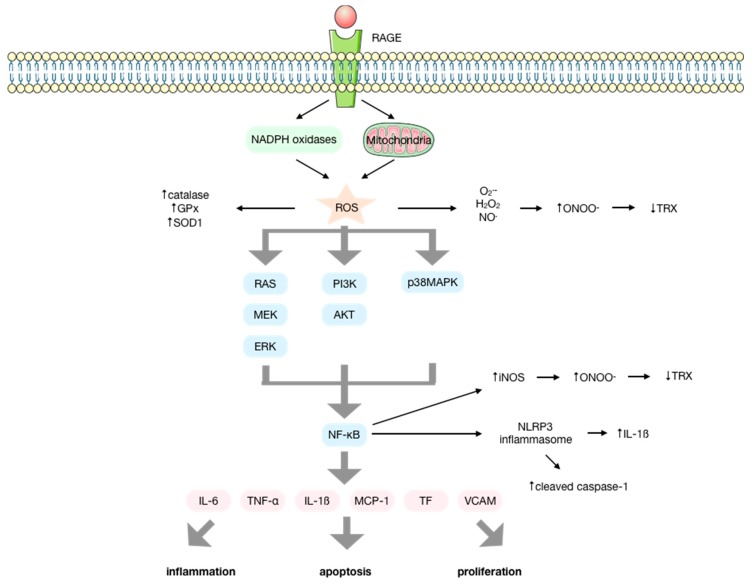
Molecular signaling pathways activated upon the interaction of advanced glycation endproducts (AGEs) with their receptor (RAGE). AGEs interact with their receptor RAGE, increasing the production of ROS by the NOX enzymes and the mitochondria, enhancing the activity of antioxidant enzymes. ROS initiate several signal transduction cascades such as RAS/MEK/ERK, IP3K/AKT or p38MAPK that lead to the activation of NF-κB that results in the activation of the NLRP-3 inflammasome and the production of several cytokines, chemokines, and other proinflammatory factors, inducing inflammation, apoptosis, and proliferation. The elevated production of O_2_^−^ and NO mediated by AGEs and the upregulation of iNOS by NF-κB favor the production of ONOO^−^ that conducts the inactivation of proteins such as thioredoxin, impeding its anti-apoptotic and antioxidant functions.

The interaction of AGEs with RAGE modulates transduction through ROS formation via NADPH oxidases and mitochondria [24,25,26,27]. This initiates several signal transduction cascades that involve p21RAS, p44/p42 mitogen-activated protein kinases (MAPK), PI3K-AKT, ERK1/2, JNK, p38, protein kinase C (PKC), and NF-κB activation [14,25,28,29,30,31,32,33,34,35,36], finally resulting in the production of cytokines, chemokines, and other proinflammatory molecules that induce inflammation [37,38,39,40,41,42], apoptosis, and proliferation [43,44,45,46,47,48]. Among these molecules, IL-6, TNF-α, IL-1β, MCP-1, tissue factor (TF), and VCAM-1 are upregulated by AGEs [22,27] (Figure 2).

AGE-RAGE interaction also results in an increased mRNA for heme oxygenase-1; it enhances the nuclear translocation of NF-κB, increases both the expression of VCAM-1 and endothelial permeability, and generates ROS by other mechanisms [25,49,50,51]. 

NLRP3 inflammasome is discussed extensively as a novel cell stress signal [52]. It is a cytosolic macromolecular complex composed of NLRP3, the adaptor protein ASC, caspase-1, and/or caspase-11 [52]. There is solid evidence that AGEs can activate NLRP3 inflammasome via oxidative and inflammatory stress [53,54,55,56]. There is an initial signal regulated by NF-κB for the transcription of NLRP3 and pro-IL-1β. After that, there is a second signal that assembles the NLRP3 inflammasome, leading to an increased protein level of cleaved caspase-1 and the maturation and secretion of the pro-inflammatory cytokine IL-1β [22,52,57].

Moreover, it has been shown that the AGE/soluble RAGE (sRAGE) ratio could be a useful biomarker for assessing the risk of developing certain diseases. Thus, an increased AGE/sRAGE ratio is an indicator of an elevated disease risk [58].

Considering the relevance of proinflammatory and prooxidant pathways in the pathogenesis of age-related diseases, AGEs are involved in relevant illnesses such as diabetes [59,60,61], insulin resistance, cardiovascular diseases [62,63], chronic renal failure [64], and neurological disorders [65,66]. 

## 3. Reactive Oxygen Species/Reactive Nitrogen Species are Intermediary Signaling Molecules of AGE Biological Activity

It has been described that AGEs can produce ROS by activating NADPH oxidases [67,68], the mitochondrial respiratory chain, microsomal enzymes, xanthine oxidase, and arachidonic acid pathways [69,70,71] by interacting with their receptor (Figure 2). There is considerable evidence that ROS are generated upon interaction of AGEs with RAGE. Studies performed in endothelial cells [25,34,49,50,51,72,73], macrophages [37], and cardiomyocytes [38] have identified an increase in the intracellular levels of H_2_O_2_, O_2_^−^, and NO, as well as a higher release of H_2_O_2_ after treatment with AGEs by using redox fluorescence probes. Thus, the activation of the AGE-RAGE axis enhances oxidative stress, affecting mitochondrial function and ultimately influencing cell metabolism under various pathological conditions [74,75]. 

Treating the cells with glycated albumin also increases the production of superoxide anions in the mitochondria. Furthermore, cells treated with AGEs showed an increase in basal oxygen consumption and proton leak as well as a reduction in the maximal respiration, spare respiration capacity, and non-mitochondrial respiration. This is in accordance with the tendency of AGEs to intensify proton leak, which is a sign of mitochondrial damage [72,76].

Moreover, AGE production of ROS also enhances the expression [77] and the activities of antioxidant enzymes such as catalase, GPx, and SOD1, although no differences are described for the activity of SOD2. However, this increase in the antioxidant defenses appears to be depleted over time [72,78], which implies an indirect effect more than a transcriptional modulation.

AGEs also produce oxidative modifications of proteins [79]. Thus, there is a production of intracellular advanced oxidation protein products (AOPPs) in endothelial cells [72] and macrophages [22] treated with AGEs. 

ROS play an essential role in mediating the RAGE signal transduction. According to this, ROS generated in the cellular milieu directly activate p21RAS [80,81], and in RAGE-bearing cells expressing a mutant form of p21RAS, suppression of the activation of ERK1/2 upon exposure to AGEs was found [31]. Furthermore, AGE-RAGE mediated activation of ERK1/2 kinases is enhanced in the presence of glutathione depletion [31]. ROS are also necessary for sustaining the phosphorylation of p38 [82] and JNK [83] caused by AGEs by inhibiting the inactivating phosphatases, allowing the activation of NF-κB [37].

AGEs are also involved in the production of reactive nitrogen species (RNS). These species are the reaction products of nitric oxide (NO) that is produced by nitric oxide synthases (NOS) and the superoxide anion (O_2_^−^) produced by NADPH oxidases [38]. Moreover, since O_2_^•–^ is enhanced by activation of NADPH oxidases mediated by AGEs, the increase in NO^•^ and O_2_^•–^ favors the production of peroxynitrite (ONOO^–^). This is an oxidizing and nitrating molecule that inactivates functional proteins [14,84]. NOS is inducible by cytokines (iNOS) so that the activation of NF-κB by AGEs finally produces an upregulation of iNOS, which can produce large amounts of NO [22,85,86,87] that leads to the induction of nitrosative stress and an increase in the levels of ONOO^−^ [88,89]. Furthermore, AGEs can also downregulate endothelial NOS (eNOS) [90,91].

The NOS inhibitor L-NMMA does not reduce intracellular H_2_O_2_, which might be explained by a lack of direct involvement of NO in the production of ROS by AGEs [26]. However, the uncoupling of eNOS can produce O_2_^−^ instead of NO [92]. In experiments with the NOS inhibitor L-NAME, it was shown that eNOS was a significant O_2_^−^ source in the presence of AGEs, which suggests that they could uncouple eNOS by enhancing ONOO^–^ production [24].

Regarding the nitration of proteins, it has been demonstrated that the activation of RAGE by AGEs induces the production of ONOO^−^, thus modulating the nitration of thioredoxin and therefore its inactivation (Figure 2). This brought, as a consequence, a loss in anti-apoptotic and antioxidant function as well as a reduced cardiac protection function in a mice model of diabetic myocardial ischemia-reperfusion injury. It has also been shown that upregulation of thioredoxin is able to inhibit the expression of RAGE, suggesting crosstalk between RAGE and thioredoxin [93,94].

## 4. AGEs Produce Reactive Oxygen Species by a NOX-Mediated Mechanism

Regarding the AGE-mediated production of ROS, some studies have also shown the activation of NADPH oxidases by AGEs in mesangial and endothelial cells [34,67,95]. There is an increase in the mRNA levels of NOX1, NOX2, NOX4, and the NADPH oxidase subunit p22phox after treating cells with AGEs [22,23,96].

AGE-mediated ROS production was inhibited after incubation with inhibitors of NADPH oxidases, such as apocynin and DPI, antioxidant enzymes such as SOD and catalase or antioxidants such as probucol and NAC. ROS production was also reduced by myxothiazol, an inhibitor of complex III of the respiratory chain. These data support the role that NADPH oxidases and mitochondria play in the intracellular production of ROS mediated by AGEs [23,24,26,72,95,96]. Moreover, when cells were pretreated with anti-RAGE or soluble (sRAGE), the expression of NOX-1, NOX-2, and NOX-4 was inhibited and hence, ROS production [23,24,26,97], thus confirming the role of the AGE-RAGE axis in ROS generation by NADPH oxidases.

It has also been proposed that crosstalk exists between NADPH oxidases and mitochondria. In that case, AGEs would trigger the production of O_2_^−^ by activating the NADPH oxidases. This O_2_^−^ would stimulate the mitochondria to produce additional superoxide [72,98]. AGEs reduced the ratio of the maximal respiration rate to a basal level and slightly increased the proton leak [72], which can decrease the production of ROS in the mitochondria [99].

The pharmacological inhibition of NADPH oxidases not only inhibited ROS production but also modulated the gene expression and transcription factor activation induced by AGEs. Thus, NADPH oxidase inhibition by DPI inhibited the expression of E-selectin [34], and apocynin inhibited the activation of NF-κB [38]. 

It has also been shown that the inhibition of NF-κB in cells treated with AGEs decreases the production of ROS since it reverts the enhancement of NOX4 expression [24]. Moreover, AGEs have also been associated with an increase in the activation of the transcription factor AP-1 [100,101] which is also involved in the activity and expression of NADPH oxidases. Thus, the inhibition of AP-1 results in a reduction of the angiotensin-II and TNF-α-dependent upregulation of NADPH oxidases and *p22phox* promoter activity [35]. These effects can be explained since NF-κB regulates three subunits of NADPH oxidase: *gp91phox, p47phox,* and *p22phox* [102,103,104], and AP-1 was implicated in the promoter activity of *p67phox* and the regulation of *p22phox* expression [35,105].

## 5. Increasing Relevance of AGEs in Diet-Related Diseases and Associated Diabetic Pathologies

Diets are currently characterized by elevated sugar intake, mainly due to the increased consumption of processed sweetened foods and drinks during the last 40 years [106]. Chronic hyperglycemia is associated with endogenous AGE formation and subsequent interaction with RAGE [3,107,108,109], which results in the initiation of numerous signaling pathways.

**Figure 3:**
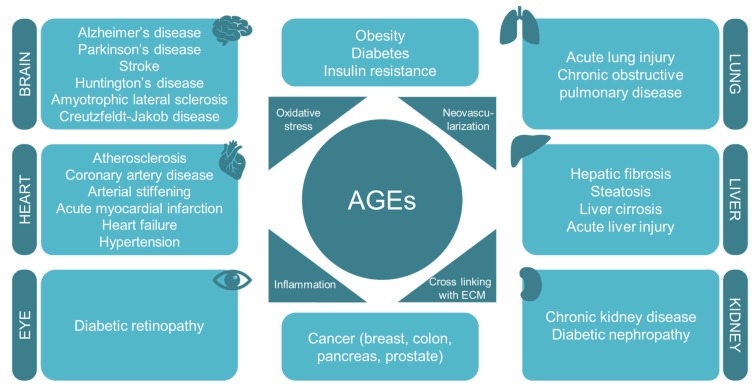
AGEs are involved in the pathogenesis of several diet-related diseases and age-associated diseases by interfering with oxidative stress, neovascularization, inflammation, and cross-linking with the extracellular matrix.

According to data from the World Health Organization, in 2016, one of the leading causes of mortality and morbidity worldwide was diabetes mellitus and its vascular complications such as atherosclerosis, diabetic nephropathy, coronary artery disease, arterial stiffening, and diabetic retinopathy [110]. Diabetes is characterized by high levels of circulating glucose [111] and increased oxidative stress [112,113,114]. A positive correlation between oxidative stress markers and glycated albumin levels has been described in patients with type 2 diabetes mellitus [96,115]. Moreover, the long-term oxidative stress produced by AGEs may result in protein damage that finally leads to endothelial dysfunction [116]. Thereby, the accumulation of AGEs has been related to diabetes and also to its associated complications [117,118,119,120,121,122,123,124,125,126] (Figure 3). However, the molecular mechanisms and the signaling pathways involved are yet to be clearly defined.

As commented above, AGEs are also associated with diabetes complications, such as insulin resistance. Hence, glycated albumin is found on the one hand to induce the expression of TNF-α, which suppresses insulin signaling [127] and, on the other hand, to impair the PI3K pathway and inhibit insulin-mediated glucose metabolism [128]. Furthermore, under hyperglycemic conditions, insulin can be directly glycated, reducing its glucose-lowering potential [129,130]. In a study in which non-obese mice were fed a diet enriched with methylglyoxal-modified albumin, there was an increase in both inflammation and oxidative stress, as well as an insulin-resistant phenotype [131]. 

It has also been found that AGEs accumulate in atherosclerotic lesions, where it is described that they contribute to endothelial dysfunction [132,133] and increase the expression of MCP-1, PAI-1, ICAM-1, and VCAM-1 [134,135,136]. AGE-associated oxidative stress appears as a central element in the pathology of atherosclerosis [137]. Moreover, AGEs decrease the expression of eNOS, diminishing the synthesis of NO, which mediates some fundamental mechanisms in endothelial dysfunction and atherosclerosis such as vasodilation or endothelial regeneration [138].

Moreover, a role for AGEs in diabetic nephropathy has been described. This diabetes complication is associated with the loss of mesangial cells in the glomerulus. It has been demonstrated that AGEs induce apoptosis and VEGF and MCP-1 expression in these cells, which contributes to an enhanced vascular permeability and correlates with hyperfiltration, proteinuria, and inflammation of the renal tissue [139].

Finally, another well-known diabetic microvascular complication is diabetic retinopathy, which is the major cause of acquired blindness. It is associated with the breakdown of the blood-retina barrier since it can produce the development of macular edema, a principal cause for vision loss in diabetes [140]. It has been demonstrated that this breakdown might be mediated by AGEs since they induce the adhesion of leukocytes to the endothelial cells of the retina and also increase the expression of ICAM-1 and DNA binding of NF-κB [141].

## 6. The Increasing Relevance of AGEs in Age-Associated Diseases

AGEs have been also associated with other important non-diabetes-related chronic disorders as a causative factor. These illnesses are, among others, hypertension, chronic kidney disease, some cardiovascular and pulmonary diseases, neurodegenerative disorders, and cancer (Figure 3).

Focusing on neurodegeneration, it must be highlighted that the brain is an organ that displays a high susceptibility to oxidative stress since it has a high rate of oxygen consumption and relatively low levels of antioxidants [142]. There is increasing evidence that oxidative stress plays an essential role in the pathogenesis of neurodegenerative disorders [143]. Furthermore, oxidative stress contributes to the formation of AGEs that, in turn, induce oxidative stress, forming a positive feedback loop that drives oxidative damage in the brain [144]. For this reason, AGEs are recognized as important players in the pathogenesis of several neurodegenerative disorders such as Alzheimer’s or Parkinson’s diseases [145,146], but also Huntington’s disease, amyotrophic lateral sclerosis or Creutzfeldt-Jakob disease [147,148].

Alzheimer’s disease is the most prevalent type of dementia in the elderly, who suffer a progressive cognitive decline, compromising their functional abilities and thus affecting their life quality [65]. In this pathological condition, the amyloid precursor protein has been found to be upregulated by AGEs [149], increasing the levels of β-amyloid, which is the principal component of senile plaques. The aggregation and deposition of β-amyloid are also accelerated by AGE-mediated crosslinking [150,151]. Moreover, ApoE might also bind to elements of the senile plaques that are modified by AGEs [152]. Regarding tau, its glycation has been showed to produce oxidative stress [153], and AGEs also contribute to the hyperphosphorylation of tau through a RAGE-mediated GSK-3β activation that will finally aggregate and form the neurofibrillary tangles [154]. 

Parkinson’s disease is the second most prevalent neurodegenerative disease [155]. It is characterized by shaking, muscle stiffness, and achiness that produce a limitation in the movement [156]. In this disease, AGEs promote the formation of Lewy bodies that contain the neurofilament α-synuclein [157]. AGEs have been found to induce the aggregation of α-synuclein, co-occurring with oxidative stress and contributing to the pathogenesis of Parkinson’s disease [158,159,160]. 

Last but not least, in the last few years, the potential implication of AGEs in carcinogenesis has drawn the attention of investigators, and the presence of AGEs has already been described in several types of cancer [161,162,163]. Moreover, it has been found that the circulating levels of AGEs were increased in patients with prostate cancer when compared with healthy patients, with the highest levels of AGEs corresponding with high-grade prostate cancer patients, according to the Gleason score [162,164,165]. 

In vitro studies have shown that AGEs promote growth, invasion, and migration of cancer cells in prostate and breast cancer [166,167]. These effects might be due to an interaction with their receptor RAGE, activating its signaling pathway since high levels of RAGE were found in tumors compared to healthy tissue [162,164,168]. Furthermore, the role of RAGE in tumor proliferation, migration, and invasion has been described [168,169], even though the molecular mechanism remains unknown and a potential mediation of ROS cannot be discarded. Silencing RAGE as a therapeutic approach produced in human prostate cancer cells an inhibition of the proliferation and a decrease in the levels of prostate-specific antigen (PSA) [170]. Moreover, in a breast cancer cell line, treatment with metformin suppressed the expression of RAGE and cell proliferation [171].

Another implication of AGEs in cancer is related to their ability to modify the extracellular matrix by establishing crosslinks that favor the invasion of cancer cells. Therefore, AGE-induced crosslinking of fibronectin promotes matrix accumulation by increasing the stiffness of collagen. In addition, the AGE-mediated crosslinking of collagen IV and laminin promotes the stiffening of the basal lamina matrix [166,172].

## 7. Use of Natural Compounds as Therapeutic Strategies

Regarding the role that AGEs play in ROS production and hence oxidative stress, and since diet is the main source of AGEs, the use of natural compounds with antioxidant properties is gaining importance. Among them, polyphenols are the most widely studied (Table 1).

One of these natural compounds is curcumin, a phenolic acid, specifically a hydrocinnamic acid, known for its antioxidant and anti-inflammatory properties [189]. It has been shown to reduce the accumulation of AGEs and the crosslinking of collagen in diabetic rats [173]. It also scavenges ROS and inhibits lipid peroxidation [174]. Furthermore, it inhibits NADPH oxidases [190] and prevents the AGE-induced increase in NF-κB and VEGF [191]. It has also been described to reduce the levels of blood glucose in type 2 diabetic mice [192], ameliorates diabetic nephropathy in rats [193], and decrease hepatosteatosis and insulin resistance in fructose-fed mice [194,195].

Some other frequently used compounds are flavonoids. These polyphenols are classified in different groups of compounds, such as flavanols. Among them, catechin, one of the main flavonoids present in green tea, reduces the formation of AGEs in diabetic rats [175], due to its anti-glycation effects dependent on its phenolic composition [176]. Catechins act as a free radical scavenger and prevent lipid peroxidation [177,196]. Authors have also shown a protective role against the oxidation-induced damage in erythrocytes in type 2 diabetes patients [197]. 

Another group of flavonoids are flavonols that include quercetin, a compound present in many foods and vegetables such as red onion or kale. It has been reported to inhibit the formation of AGEs in a dose-dependent manner by trapping the dicarbonyls glyoxal and methylglyoxal and to suppress their induced protein glycation [179]. Moreover, extracts from the leaves of the luobuma plant, enriched in catechin, quercetin, and rutin, have antioxidant and anti-inflammatory properties [198] and can inhibit lipid peroxidation and the formation of AGEs [178,199]. Other flavonols include kaempferol, found in high amounts in garlic. It has the ability to inhibit the formation of AGEs as well as the crosslinking of proteins [180]. 

In addition, we can also find flavonoids in citrus fruits and tomatoes, as the flavanone naringenin, characterized by its antioxidant, anti-inflammatory, antidiabetic, and antiatherogenic properties [200]. Naringenin has also shown the capacity of inhibiting AGE formation and AGE-induced cellular oxidative stress and inflammation, reducing the expression of genes such as TNF-α, IL-1β, and COX-2 as well as the production of ROS [181]. Another flavanone, hesperetin, the aglycone of the flavanone hesperidin, present in lemons and sweet oranges, has been shown to upregulate glyoxalase-1 and to inhibit the AGE/RAGE axis by decreasing the formation of AGEs as well as the protein levels of RAGE, thus reducing inflammation and decreasing the levels of IL-1β and TNF-α [182].

Moreover, apigenin, belonging to the flavone group and mostly found in parsley, celery, and chamomile tea, was found to suppress AGE-induced ROS production and decrease the levels of proinflammatory cytokines and adhesion molecules. This effect was likely mediated by the downregulation of RAGE, p-ERK 1/2, and NF-κB and a reduced NOX activity that subsequently led to an upregulation of NRF2 and antioxidant defenses [183].

Furthermore, the isoflavone genistein, found in lupin, fava beans, and soybeans, has been very recently tested in vivo. It has been shown to inhibit AGE formation, downregulate RAGE, and upregulate the expression of glyoxalase-1 and 2 in mice fed a high-fat diet [184].

Another phenolic phytochemical, hydroxytyrosol, a phenylethanoid found in olive leaves and olive oil, reduced the production of AGEs, showed a high methylglyoxal-trapping capacity, and attenuated protein carbonylation in the hepatic cell line HepG2 [185].

Finally, some studies have been carried out using resveratrol. This is a non-flavonoid polyphenolic compound present in grapes, red wine, berries, and peanuts. It has antioxidant, anti-inflammatory, anti-aging, and cardioprotective characteristics [201]. It ameliorates hyperglycemia, hyperlipidemia, and diabetic complications [202], mostly by activating SIRT-1 and AMPK [203]. A recent study concluded that treatment with resveratrol decreases the levels of protein carbonyls, AOPPs, ROS, and AGEs in plasma and the liver [186,187,188].

## 8. Conclusions

The implication of diet in AGE formation and the consequent role that AGEs play in oxidative stress, inflammation, and protein modification are irrefutable. However, there are still so many unanswered questions, and the molecular mechanisms by which AGEs participate in the pathogenesis of such a wide variety of diseases have yet to be further studied. It has been proposed that dietary AGE restriction and an increased dietary awareness could be useful in restraining AGE accumulation, thus providing a potential approach for chronic disease prevention [204].

One of the main challenges to be confronted is the fact that AGEs are a heterogeneous group of different molecules, with different chemical structures, and not all of them might exert the same molecular effects. The immense majority of the studies published to date used glycated albumin or a mixture of different AGEs to perform the experiments. These compounds were usually produced in their laboratories following non-reproducible methodologies. Thus, results obtained between different laboratories are hardly comparable.

In order to solve these difficulties, our group, as part of the European consortium SALIVAGES, within the framework of the Joint Programming Initiative “A Healthy Diet for a Healthy Life”, investigates the preclinical characterization of AGEs and dicarbonyls to find out the relevance of particular AGEs as novel biomarkers for diet-related diseases.

## Figures and Tables

**Table 1:** List of some of the natural antioxidant compounds that have been used in the AGE field and the AGE-related effect that they produce.

Molecule	Type of Compound	Found in	AGE-Related Effect	References
Curcumin	Hydrocinnamic acid	*Curcuma longa* plants	↓AGE accumulation ↓crosslinking of collagen prevents AGE-induced increase in NF-κB and VEGF	[173,174]
Catechin	Flavanol	Green tea	↓AGE formation	[175,176,177,178]
Quercetin	Flavonol	Red onion, kale	↓AGE formation suppresses dicarbonyls-induced protein glycation	[178,179]
Kaempferol	Flavonol	Garlic	↓AGE formation ↓protein crosslinking	[180]
Naringenin	Flavanone	Citrus fruits, tomatoes	inhibits AGE formation inhibits AGE-induced oxidative stress and inflammation	[181]
Hesperetin	Flavanone	Lemons, sweet oranges	↑glyoxalase-1 ↓AGE formation ↓RAGE protein levels inhibits AGE/RAGE axis	[182]
Apigenin	Flavone	Parsley, celery, chamomile tea	inhibits AGE-induced ROS production ↓RAGE protein levels	[183]
Genistein	Isoflavone	Lupin, fava beans, soybeans	inhibits AGE formation ↓RAGE protein levels ↑glyoxalase-1 and 2	[184]
Hydroxytyrosol	Phenylethanoid	Olive leaves and olive oil	↓AGE formation ↓protein carbonylation	[185]
Resveratrol	Stilbene	Grapes, red wine, berries, peanuts	↓AGE formation ↓protein carbonylation ↓AOPP	[186,187,188]

## References

[1] Nursten H. (2005). The Maillard Reaction: Chemistry, Biochemistry and Implications.

[2] Vistoli G., De Maddis D., Cipak A., Zarkovic N., Carini M., Aldini G. (2013). Advanced glycoxidation and lipoxidation end products (AGEs and ALEs): An overview of their mechanisms of formation. Free Radic. Res..

[3] Vlassara H., Uribarri J. (2014). Advanced glycation end products (AGE) and diabetes: Cause, effect, or both?. Curr. Diab. Rep..

[4] Poulsen M.W., Hedegaard R.V., Andersen J.M., de Courten B., Bügel S., Nielsen J., Skibsted L.H., Dragsted L.O. (2013). Advanced glycation endproducts in food and their effects on health. Food Chem. Toxicol..

[5] Vlassara H., Cai W., Tripp E., Pyzik R., Yee K., Goldberg L., Tansman L., Chen X., Mani V., Fayad Z.A. (2016). Oral AGE restriction ameliorates insulin resistance in obese individuals with the metabolic syndrome: A randomised controlled trial. Diabetologia.

[6] Aragno M., Mastrocola R. (2017). Dietary sugars and endogenous formation of advanced glycation endproducts: Emerging mechanisms of disease. Nutrients.

[7] Bunn H.F., Higgins P.J. (1981). Reaction of monosaccharides with proteins: Possible evolutionary significance. Science.

[8] Rippe J.M., Angelopoulos T.J. (2013). Sucrose, high-fructose corn syrup, and fructose, their metabolism and potential health effects: What do we really know?. Adv. Nutr..

[9] Mastrocola R., Collino M., Rogazzo M., Medana C., Nigro D., Boccuzzi G., Aragno M. (2013). Advanced glycation end products promote hepatosteatosis by interfering with SCAP-SREBP pathway in fructose-drinking mice. Am. J. Physiol. Liver Physiol..

[10] Lee O., Bruce W.R., Dong Q., Bruce J., Mehta R., O’Brien P.J. (2009). Fructose and carbonyl metabolites as endogenous toxins. Chem. Biol. Interact..

[11] Jono T., Miyazaki A., Nagai R., Sawamura T., Kitamura T., Horiuchi S. (2002). Lectin-like oxidized low density lipoprotein receptor-1 (LOX-1) serves as an endothelial receptor for advanced glycation end products (AGE). FEBS Lett..

[12] Ohgami N., Nagai R., Ikemoto M., Arai H., Kuniyasu A., Horiuchi S., Nakayama H. (2001). CD36, a Member of the Class B Scavenger Receptor Family, as a Receptor for Advanced Glycation End Products. J. Biol. Chem..

[13] Ramasamy R., Yan S.F., Schmidt A.M. (2011). Receptor for AGE (RAGE): Signaling mechanisms in the pathogenesis of diabetes and its complications. Ann. N. Y. Acad. Sci..

[14] Ott C., Jacobs K., Haucke E., Navarrete Santos A., Grune T., Simm A. (2014). Role of advanced glycation end products in cellular signaling. Redox Biol..

[15] Neeper M., Schmidt A.M., Brett J., Yan S.D., Wang F., Pan Y.C., Elliston K., Stern D., Shaw A. (1992). Cloning and expression of a cell surface receptor for advanced glycosylation end products of proteins. J. Biol. Chem..

[16] Schmidt A.M., Vianna M., Gerlach M., Brett J., Ryan J., Kao J., Esposito C., Hegarty H., Hurley W., Clauss M. (1992). Isolation and characterization of two binding proteins for advanced glycosylation end products from bovine lung which are present on the endothelial cell surface. J. Biol. Chem..

[17] Schmidt A.M., Yan S.D., Wautier J.L., Stern D. (1999). Activation of receptor for advanced glycation end products: A mechanism for chronic vascular dysfunction in diabetic vasculopathy and atherosclerosis. Circ. Res..

[18] Nedić O., Rattan S.I.S., Grune T., Trougakos I.P. (2013). Molecular effects of advanced glycation end products on cell signalling pathways, ageing and pathophysiology. Free Radic. Res..

[19] Yang J., Zhang F., Shi H., Gao Y., Dong Z., Ma L., Sun X., Li X., Chang S., Wang Z. (2019). Neutrophil-derived advanced glycation end products-Nε-(carboxymethyl) lysine promotes RIP3-mediated myocardial necroptosis via RAGE and exacerbates myocardial ischemia/reperfusion injury. FASEB J..

[20] Wang Y., Xu H., Liu X., Liu L., Wu Y., Gong Z.Y. (2019). Studies on mechanism of free Nε-(carboxymethyl)lysine-induced toxic injury in mice. J. Biochem. Mol. Toxicol..

[21] Wu Y., Li Y., Zheng L., Wang P., Liu Y., Wu Y., Gong Z. (2019). The neurotoxicity of Nε-(carboxymethyl)lysine in food processing by a study based on animal and organotypic cell culture. Ecotoxicol. Environ. Saf..

[22] Yu W., Tao M., Zhao Y., Hu X., Wang M. (2018). 4′-methoxyresveratrol alleviated AGE-induced inflammation via RAGE-mediated NF-κB and NLRP3 inflammasome pathway. Molecules.

[23] Chen Y.-H., Chen Z.-W., Li H.-M., Yan X.-F., Feng B. (2018). AGE/RAGE-Induced EMP Release via the NOX-Derived ROS Pathway. J. Diabetes Res..

[24] Rodiño-Janeiro B.K., Paradela-Dobarro B., Raposeiras-Roubín S., González-Peteiro M., González-Juanatey J.R., Álvarez E. (2015). Glycated human serum albumin induces NF-κB activation and endothelial nitric oxide synthase uncoupling in human umbilical vein endothelial cells. J. Diabetes Complicat..

[25] Yan S.D., Schmidt A.M., Anderson G.M., Zhang J., Brett J., Zou Y.S., Pinsky D., Stern D. (1994). Enhanced cellular oxidant stress by the interaction of advanced glycation end products with their receptors/binding proteins. J. Biol. Chem..

[26] Wautier M.-P., Chappey O., Corda S., Stern D.M., Schmidt A.M., Wautier J.L. (2001). Activation of NADPH oxidase by AGE links oxidant stress to altered gene expression via RAGE. Am. J. Physiol. Metab..

[27] Matheny H.E., Deem T.L., Cook-Mills J.M. (2000). Lymphocyte migration through monolayers of endothelial cell lines involves VCAM-1 signaling via endothelial cell NADPH oxidase. J. Immunol..

[28] Park S., Ahn J.Y., Lim M.J., Kim M.H., Yun Y.S., Jeong G., Song J.Y. (2010). Sustained expression of NADPH oxidase 4 by p38 MAPK-AKT signaling potentiates radiation-induced differentiation of lung fibroblasts. J. Mol. Med..

[29] Hofmann M.A., Drury S., Fu C., Qu W., Taguchi A., Lu Y., Avila C., Kambham N., Bierhaus A., Nawroth P. (1999). RAGE mediates a novel proinflammatory axis: A central cell surface receptor for S100/calgranulin polypeptides. Cell.

[30] Huttunen H.J., Fages C., Rauvala H. (1999). Receptor for advanced glycation end products (RAGE)-mediated neurite outgrowth and activation of NF-κB require the cytoplasmic domain of the receptor but different downstream signaling pathways. J. Biol. Chem..

[31] Lander H.M., Tauras J.M., Ogiste J.S., Hori O., Moss R.A., Schmidt A.M. (1997). Activation of the Receptor for Advanced Glycation End Products Triggers a p21 ras -dependent Mitogen-activated Protein Kinase Pathway Regulated by Oxidant Stress. J. Biol. Chem..

[32] Taguchi A., Blood D.C., del Toro G., Canet A., Lee D.C., Qu W., Tanji N., Lu Y., Lalla E., Fu C. (2000). Blockade of RAGE-amphoterin signalling suppresses tumour growth and metastases. Nature.

[33] Xie J., Méndez J.D., Méndez-Valenzuela V., Aguilar-Hernández M.M. (2013). Cellular signalling of the receptor for advanced glycation end products (RAGE). Cell. Signal..

[34] Higai K., Shimamura A., Matsumoto K. (2006). Amadori-modified glycated albumin predominantly induces E-selectin expression on human umbilical vein endothelial cells through NADPH oxidase activation. Clin. Chim. Acta.

[35] Manea A., Manea S.A., Gafencu A.V., Raicu M., Simionescu M. (2008). AP-1-dependent transcriptional regulation of NADPH oxidase in human aortic smooth muscle cells: Role of p22phox subunit. Arterioscler. Thromb. Vasc. Biol..

[36] Manea A., Tanase L.I., Raicu M., Simionescu M. (2010). Transcriptional regulation of NADPH oxidase isoforms, Nox1 and Nox4, by nuclear factor-κB in human aortic smooth muscle cells. Biochem. Biophys. Res. Commun..

[37] Cohen M.P., Shea E., Chen S., Shearman C.W. (2003). Glycated albumin increases oxidative stress, activates NF-κB and extracellular signal-regulated kinase (ERK), and stimulates erk-dependent transforming growth factor-β1 production in macrophage RAW cells. J. Lab. Clin. Med..

[38] Zhang M., Kho A.L., Anilkumar N., Chibber R., Pagano P.J., Shah A.M., Cave A.C. (2006). Glycated Proteins Stimulate Reactive Oxygen Species Production in Cardiac Myocytes. Circulation.

[39] Hattori Y., Banba N., Gross S.S., Kasai K. (1999). Glycated serum albumin-induced nitric oxide production in vascular smooth muscle cells by nuclear factor κB-dependent transcriptional activation of inducible nitric oxide synthase. Biochem. Biophys. Res. Commun..

[40] Nevado J., Peiró C., Vallejo S., El-Assar M., Lafuente N., Matesanz N., Azcutia V., Cercas E., Sánchez-Ferrer C.F., Rodríguez-Mañas L. (2005). Amadori adducts activate nuclear factor-κB-related proinflammatory genes in cultured human peritoneal mesothelial cells. Br. J. Pharmacol..

[41] Zhang Q., Lenardo M.J., Baltimore D. (2017). 30 Years of NF-κB: A Blossoming of Relevance to Human Pathobiology. Cell.

[42] Yeh C.H., Sturgis L., Haidacher J., Zhang X.N., Sherwood S.J., Bjercke R.J., Juhasz O., Crow M.T., Tilton R.G., Denner L. (2001). Requirement for p38 and p44/p42 mitogen-activated protein kinases in RAGE-mediated nuclear factor-kappaB transcriptional activation and cytokine secretion. Diabetes.

[43] Wautier M.P., Guillausseau P.J., Wautier J.L. (2017). Activation of the receptor for advanced glycation end products and consequences on health. Diabetes Metab. Syndr. Clin. Res. Rev..

[44] Luevano-Contreras C., Chapman-Novakofski K. (2010). Dietary advanced glycation end products and aging. Nutrients.

[45] Gaens K.H., Stehouwer C.D., Schalkwijk C.C. (2010). The Nε-(carboxymethyl)lysine-RAGE axis: Putative implications for the pathogenesis of obesity-related complications. Expert Rev. Endocrinol. Metab..

[46] Oeckinghaus A., Hayden M.S., Ghosh S. (2011). Crosstalk in NF-κB signaling pathways. Nat. Immunol..

[47] Alikhani M., Alikhani Z., Boyd C., MacLellan C.M., Raptis M., Liu R., Pischon N., Trackman P.C., Gerstenfeld L., Graves D.T. (2007). Advanced glycation end products stimulate osteoblast apoptosis via the MAP kinase and cytosolic apoptotic pathways. Bone.

[48] Meng H.Z., Zhang W.L., Liu F., Yang M.W. (2015). Advanced glycation end products affect osteoblast proliferation and function by modulating autophagy via the receptor of advanced glycation end products/raf protein/mitogen-activated protein kinase/extracellular signalregulated kinase kinase/extracellular. J. Biol. Chem..

[49] Schmidt A.M., Hori O., Xian J., Li J.F., Crandall J., Zhang J., Cao R., Yan S.D., Brett J., Stern D. (1995). Advanced glycation endproducts interacting with their endothelial receptor induce expression of vascular cell adhesion molecule-(VCAM-1) in cultured human endothelial cells and in mice. J. Clin. Investig..

[50] Wautier J.L., Wautier M.P., Schmidt A.M., Anderson G.M., Hori O., Zoukourian C., Capron L., Chappey O., Yan S.D., Brett J. (1994). Advanced glycation end products (AGEs) on the surface of diabetic erythrocytes bind to the vessel wall via a specific receptor inducing oxidant stress in the vasculature: A link between surface-associated AGEs and diabetic complications. Proc. Natl. Acad. Sci. USA.

[51] Wautier J.L., Zoukourian C., Chappey O., Wautier M.P., Guillausseau P.J., Cao R., Hori O., Stern D., Schmidt A.M. (1996). Receptor-mediated endothelial cell dysfunction in diabetic vasculopathy: Soluble receptor for advanced glycation end products blocks hyperpermeability in diabetic rats. J. Clin. Investig..

[52] Abderrazak A., Syrovets T., Couchie D., El Hadri K., Friguet B., Simmet T., Rouis M. (2015). NLRP3 inflammasome: From a danger signal sensor to a regulatory node of oxidative stress and inflammatory diseases. Redox Biol..

[53] Yeh W., Yang H., Pai M., Wu C., Chen J. (2017). Long-term administration of advanced glycation end-product stimulates the activation of NLRP3 inflammasome and sparking the development of renal injury. J. Nutr. Biochem..

[54] Song Y., Wang Y., Zhang Y., Geng W., Liu W., Gao Y., Li S., Wang K., Wu X., Kang L. (2017). Advanced glycation end products regulate anabolic and catabolic activities via NLRP3-inflammasome activation in human nucleus pulposus cells. J. Cell. Mol. Med..

[55] Kong X., Lu A., Yao X., Hua Q., Li X., Qin L., Zhang H., Meng G., Su Q. (2017). Activation of NLRP3 Inflammasome by Advanced Glycation End Products Promotes Pancreatic Islet Damage. Oxid. Med. Cell. Longev..

[56] Deng X., Huang W., Peng J., Zhu T.T., Sun X.L., Zhou X.Y., Yang H., Xiong J.F., He H.Q., Xu Y.H. (2018). Irisin Alleviates Advanced Glycation End Products-Induced Inflammation and Endothelial Dysfunction via Inhibiting ROS-NLRP3 Inflammasome Signaling. Inflammation.

[57] Vanaja S.K., Rathinam V.A., Fitzgerald K.A. (2015). Mechanisms of inflammasome activation: Recent advances and novel insights. Trends Cell Biol..

[58] Prasad K. (2019). Is there any evidence that AGE/sRAGE is a universal biomarker/risk marker for diseases?. Mol. Cell. Biochem..

[59] Baynes J.W. (2003). Chemical modification of protein by lipids in diabetes. Clin. Chem. Lab. Med..

[60] Yamagishi S.I., Maeda S., Matsui T., Ueda S., Fukami K., Okuda S. (2012). Role of advanced glycation end products (AGEs) and oxidative stress in vascular complications in diabetes. Biochim. Biophys. Acta Gen. Subj..

[61] Yap F.Y., Kantharidis P., Coughlan M.T., Slattery R., Forbes J.M. (2012). Advanced glycation end products as environmental risk factors for the development of type 1 diabetes. Curr. Drug Targets.

[62] Fishman S.L., Sonmez H., Basman C., Singh V., Poretsky L. (2018). The role of advanced glycation end-products in the development of coronary artery disease in patients with and without diabetes mellitus: A review. Mol. Med..

[63] Yuan T., Yang T., Chen H., Fu D., Hu Y., Wang J., Yuan Q., Yu H., Xu W., Xie X. (2019). New insights into oxidative stress and inflammation during diabetes mellitus-accelerated atherosclerosis. Redox Biol..

[64] Rabbani N., Thornalley P.J. (2018). Advanced glycation end products in the pathogenesis of chronic kidney disease. Kidney Int..

[65] Li J., Liu D., Sun L., Lu Y., Zhang Z. (2012). Advanced glycation end products and neurodegenerative diseases: Mechanisms and perspective. J. Neurol. Sci..

[66] Muronetz V.I., Melnikova A.K., Seferbekova Z.N., Barinova K.V., Schmalhausen E.V. (2017). Glycation, glycolysis, and neurodegenerative diseases: Is there any connection?. Biochemistry.

[67] Basta G., Lazzerini G., Del Turco S., Ratto G.M., Schmidt A.M., De Caterina R. (2005). At least 2 distinct pathways generating reactive oxygen species mediate vascular cell adhesion molecule-1 induction by advanced glycation end products. Arterioscler. Thromb. Vasc. Biol..

[68] Nam M.H., Son W.R., Lee Y.S., Lee K.W. (2015). Glycolaldehyde-derived advanced glycation end products (glycol-AGEs)-induced vascular smooth muscle cell dysfunction is regulated by the AGES-receptor (RAGE) axis in endothelium. Cell Commun. Adhes..

[69] Serron S.C., Dwivedi N., Backes W.L. (2000). Ethylbenzene induces microsomal oxygen free radical generation: Antibody-directed characterization of the responsible cytochrome P450 enzymes. Toxicol. Appl. Pharmacol..

[70] Suzuki K., Yamamoto T., Sato A., Murayama T., Amitani R., Yamamoto K., Kuze F. (1993). Lipopolysaccharide primes human alveolar macrophages for enhanced release of superoxide anion and leukotriene B4: Self-limitations of the priming response with protein synthesis. Am. J. Respir. Cell Mol. Biol..

[71] Wüllner U., Seyfried J., Groscurth P., Beinroth S., Winter S., Gleichmann M., Heneka M., Löschmann P.A., Schulz J.B., Weller M. (1999). Glutathione depletion and neuronal cell death: The role of reactive oxygen intermediates and mitochondrial function. Brain Res..

[72] Dobi A., Bravo S.B., Veeren B., Paradela-Dobarro B., Álvarez E., Meilhac O., Viranaicken W., Baret P., Devin A., Rondeau P. (2019). Advanced glycation end-products disrupt human endothelial cells redox homeostasis: New insights into reactive oxygen species production. Free Radic. Res..

[73] Yu W., Hu X., Wang M. (2018). Pterostilbene inhibited advanced glycation end products (AGEs)-induced oxidative stress and inflammation by regulation of RAGE/MAPK/NF-κB in RAW264.7 cells. J. Funct. Foods.

[74] Wang X.L., Yu T., Yan Q.C., Wang W., Meng N., Li X.J., Luo Y.H. (2015). AGEs Promote Oxidative Stress and Induce Apoptosis in Retinal Pigmented Epithelium Cells RAGE-dependently. J. Mol. Neurosci..

[75] Boyer F., Vidot J.B., Dubourg A.G., Rondeau P., Essop M.F., Bourdon E. (2015). Oxidative Stress and Adipocyte Biology: Focus on the Role of AGEs. Oxid. Med. Cell. Longev..

[76] Li Y., Chang Y., Ye N., Chen Y., Zhang N., Sun Y. (2017). Advanced glycation end products-induced mitochondrial energy metabolism dysfunction alters proliferation of human umbilical vein endothelial cells. Mol. Med. Rep..

[77] Lee B.W., Ihm J., Kang J.G., Cho M.G., Yoo H.J., Ihm S.H. (2007). Amadori-glycated albumin-induced vascular smooth muscle cell proliferation and expression of inhibitor of apoptosis protein-1 and nerve growth factor-γ. Biofactors.

[78] Ren X., Ren L., Wei Q., Shao H., Chen L., Liu N. (2017). Advanced glycation end-products decreases expression of endothelial nitric oxide synthase through oxidative stress in human coronary artery endothelial cells. Cardiovasc. Diabetol..

[79] Singh N.R., Rondeau P., Hoareau L., Bourdon E. (2007). Identification of preferential protein targets for carbonylation in human mature adipocytes treated with native or glycated albumin. Free Radic. Res..

[80] Lander H.M., Hajjar D.P., Hempstead B.L., Mirza U.A., Chait B.T., Campbell S., Quilliam L.A. (1997). A Molecular Redox Switch on p21 ras. J. Biol. Chem..

[81] Lander H.M., Milbank A.J., Tauras J.M., Hajjar D.P., Hempstead B.L., Schartz G.D., Kraemer R.T., Mirza U.A., Chait B.T., Burk S.C. (1996). Redox regulation of cell signalling. Nature.

[82] Lan A., Xu W., Zhang H., Hua X., Zheng D., Guo R., Shen N., Hu F., Feng J., Liu D. (2013). Inhibition of ROS-activated p38MAPK pathway is involved in the protective effect of H2S against chemical hypoxia-induced inflammation in PC12 cells. Neurochem. Res..

[83] Kamata H., Honda S.I., Maeda S., Chang L., Hirata H., Karin M. (2005). Reactive oxygen species promote TNFα-induced death and sustained JNK activation by inhibiting MAP kinase phosphatases. Cell.

[84] San Martin A., Foncea R., Laurindo F.R., Ebensperger R., Griendling K.K., Leighton F. (2007). Nox1-based NADPH oxidase-derived superoxide is required for VSMC activation by advanced glycation end-products. Free Radic. Biol. Med..

[85] Amore A., Cirina P., Conti G., Cerutti F., Bagheri N., Emancipator S.N., Coppo R. (2004). Amadori-configurated albumin induces nitric oxide-dependent apoptosis of endothelial cells: A possible mechanism of diabetic vasculopathy. Nephrol. Dial. Transplant..

[86] Liu Y., Ma Y., Wang R., Xia C., Zhang R., Lian K., Luan R., Sun L., Yang L., Lau W.B. (2010). Advanced Glycation End Products Accelerate Ischemia/Reperfusion Injury through Receptor of Advanced End Product/Nitrative Thioredoxin Inactivation in Cardiac Microvascular Endothelial Cells. Antioxid. Redox Signal..

[87] Amore A., Cirina P., Mitola S., Peruzzi L., Gianoglio B., Rabbone I., Sacchetti C., Cerutti F., Grillo C., Coppo R. (1997). Nonenzymatically glycated albumin (Amadori adducts) enhances nitric oxide synthase activity and gene expression in endothelial cells. Kidney Int..

[88] Mizutani K., Ikeda K., Ito T., Tamaki K., Nara Y., Yamori Y. (2000). Protective effect of inducible type nitric oxide synthase against intracellular oxidative stress caused by advanced glycation end-products in vascular smooth muscle cells from stroke-prone spontaneously hypertensive rats. J. Hypertens..

[89] Wong A., Dukic-Stefanovic S., Gasic-Milenkovic J., Schinzel R., Wiesinger H., Riederer P., Münch G. (2001). Anti-inflammatory antioxidants attenuate the expression of inducible nitric oxide synthase mediated by advanced glycation endproducts in murine microglia. Eur. J. Neurosci..

[90] Talmor Y., Golan E., Benchetrit S., Bernheim J., Klein O., Green J., Rashid G. (2008). Calcitriol blunts the deleterious impact of advanced glycation end products on endothelial cells. Am. J. Physiol. Physiol..

[91] Xu B., Ji Y., Yao K., Cao Y.-X., Ferro A. (2005). Inhibition of human endothelial cell nitric oxide synthesis by advanced glycation end-products but not glucose: Relevance to diabetes. Clin. Sci..

[92] Förstermann U. (2010). Nitric oxide and oxidative stress in vascular disease. Pflugers Arch. Eur. J. Physiol..

[93] Liu Y., Qu Y., Wang R., Ma Y., Xia C., Gao C., Liu J., Lian K., Xu A., Lu X. (2012). The alternative crosstalk between RAGE and nitrative thioredoxin inactivation during diabetic myocardial ischemia-reperfusion injury. Am. J. Physiol. Metab..

[94] Ren X., Wang N.N., Qi H., Qiu Y.Y., Zhang C.H., Brown E., Kong H., Kong L. (2018). Up-regulation thioredoxin inhibits advanced glycation end products-induced neurodegeneration. Cell. Physiol. Biochem..

[95] Yoo C.W., Song C.Y., Kim B.C., Hong H.K., Lee H.S. (2004). Glycated Albumin Induces Superoxide Generation in Mesangial Cells. Cell. Physiol. Biochem..

[96] Rodiño-Janeiro B.K., González-Peteiro M., Ucieda-Somoza R., González-Juanatey J.R., Álvarez E. (2010). Glycated albumin, a precursor of advanced glycation end-products, up-regulates NADPH oxidase and enhances oxidative stress in human endothelial cells: Molecular correlate of diabetic vasculopathy. Diabetes Metab. Res. Rev..

[97] Downs C.A., Kreiner L.H., Johnson N.M., Brown L.A., Helms M.N. (2015). Receptor for advanced glycation end-products regulates lung fluid balance via protein kinase C-gp91phox signaling to epithelial sodium channels. Am. J. Respir. Cell Mol. Biol..

[98] Doughan A.K., Harrison D.G., Dikalov S.I. (2008). Molecular Mechanisms of Angiotensin II–Mediated Mitochondrial Dysfunction. Circ. Res..

[99] Zorov D.B., Juhaszova M., Sollott S.J. (2014). Mitochondrial Reactive Oxygen Species (ROS) and ROS-Induced ROS Release. Physiol. Rev..

[100] Hattori Y., Suzuki M., Hattori S., Kasai K. (2002). Vascular smooth muscle cell activation by glycated albumin (Amadori adducts). Hypertension.

[101] Okumura A., Mitamura Y., Namekata K., Nakamura K., Harada C., Harada T. (2007). Glycated albumin induces activation of activator protein-1 in retinal glial cells. Jpn. J. Ophthalmol..

[102] Pierce G.L., Lesniewski L.A., Lawson B.R., Beske S.D., Seals D.R. (2009). Nuclear factor-κB activation contributes to vascular endothelial dysfunction via oxidative stress in overweight/obese middle-aged and older humans. Circulation.

[103] Anrather J., Racchumi G., Iadecola C. (2006). NF-κB regulates phagocytic NADPH oxidase by inducing the expression of gp91phox. J. Biol. Chem..

[104] Manea A., Manea S.A., Gafencu A.V., Raicu M. (2007). Regulation of NADPH oxidase subunit p22phox by NF-kB in human aortic smooth muscle cells. Arch. Physiol. Biochem..

[105] Gauss K.A., Bunger P.L., Quinn M.T. (2002). AP-1 is essential for p67 phox promoter activity. J. Leukoc. Biol..

[106] McCrory M.A., Shaw A.C., Lee J.A. (2016). Energy and Nutrient Timing for Weight Control: Does Timing of Ingestion Matter?. Endocrinol. Metab. Clin. N. Am..

[107] Hanssen N.M., Beulens J.W., Van Dieren S., Scheijen J.L., Spijkerman A.M., van der Schouw Y.T., Stehouwer C.D., Schalkwijk C.G. (2015). Plasma advanced glycation end products are associated with incident cardiovascular events in individuals with type 2 diabetes: A case-cohort study with a median follow-up of 10 years (EPIC-NL). Diabetes.

[108] Singh V.P., Bali A., Singh N., Jaggi A.S. (2014). Advanced glycation end products and diabetic complications. Korean J. Physiol. Pharmacol..

[109] Higgins P.J., Bunn H.F. (1981). Kinetic analysis of the nonenzymatic glycosylation of hemoglobin. J. Biol. Chem..

[110] Deshpande A.D., Harris-Hayes M., Schootman M. (2008). Epidemiology of diabetes and diabetes-related complications. Phys. Ther..

[111] Yamagishi S.I., Matsui T. (2016). Pathologic role of dietary advanced glycation end products in cardiometabolic disorders, and therapeutic intervention. Nutrition.

[112] Baynes J.W., Thorpe S.R. (1999). Role of Oxidative Stress in Diabetic Complications. Diabetes.

[113] De M Bandeira S., da Fonseca L.J., da S Guedes G., Rabelo L.A., Goulart M.O., Vasconcelos S.M. (2013). Oxidative stress as an underlying contributor in the development of chronic complications in diabetes mellitus. Int. J. Mol. Sci..

[114] Tabit C.E., Chung W.B., Hamburg N.M., Vita J.A. (2010). Endothelial dysfunction in diabetes mellitus: Molecular mechanisms and clinical implications. Rev. Endocr. Metab. Disord..

[115] Nojima H., Watanabe H., Yamane K., Kitahara Y., Sekikawa K., Yamamoto H., Yokoyama A., Inamizu T., Asahara T., Kohno N. (2008). Effect of aerobic exercise training on oxidative stress in patients with type 2 diabetes mellitus. Metabolism.

[116] Patche J., Girard D., Catan A., Boyer F., Dobi A., Planesse C., Diotel N., Guerin-Dubourg A., Baret P., Bravo S.B. (2017). Diabetes-induced hepatic oxidative stress: A new pathogenic role for glycated albumin. Free Radic. Biol. Med..

[117] Cohen M.P., Ziyadeh F.N., Chen S. (2006). Amadori-modified glycated serum proteins and accelerated atherosclerosis in diabetes: Pathogenic and therapeutic implications. J. Lab. Clin. Med..

[118] Meerwaldt R., Links T., Zeebregts C., Tio R., Hillebrands J.L., Smit A. (2008). The clinical relevance of assessing advanced glycation endproducts accumulation in diabetes. Cardiovasc. Diabetol..

[119] Lu L., Pu L.J., Zhang Q., Wang L.J., Kang S., Zhang R.Y., Chen Q.J., Wang J.G., De Caterina R., Shen W.F. (2009). Increased glycated albumin and decreased esRAGE levels are related to angiographic severity and extent of coronary artery disease in patients with type 2 diabetes. Atherosclerosis.

[120] Kumeda Y., Inaba M., Shoji S., Ishimura E., Inariba H., Yabe S., Okamura M., Nishizawa Y. (2008). Significant correlation of glycated albumin, but not glycated haemoglobin, with arterial stiffening in haemodialysis patients with type 2 diabetes. Clin. Endocrinol..

[121] Orasanu G., Plutzky J. (2009). The Pathologic Continuum of Diabetic Vascular Disease. J. Am. Coll. Cardiol..

[122] Fosmark D.S., Torjesen P.A., Kilhovd B.K., Berg T.J., Sandvik L., Hanssen K.F., Agardh C.D., Agardh E. (2006). Increased serum levels of the specific advanced glycation end product methylglyoxal-derived hydroimidazolone are associated with retinopathy in patients with type 2 diabetes mellitus. Metabolism.

[123] Aso Y., Inukai T., Tayama K., Takemura Y. (2000). Serum concentrations of advanced glycation endproducts are associated with the development of atherosclerosis as well as diabetic microangiopathy in patients with type 2 diabetes. Acta Diabetol..

[124] Singh R., Barden A., Mori T., Beilin L. (2001). Advanced glycation end-products: A review. Diabetologia.

[125] Brownlee M., Cerami A., Vlassara H. (1988). Advanced glycosylation end products in tissue and the biochemical basis of diabetic complications. N. Engl. J. Med..

[126] Fukushima Y., Daida H., Morimoto T., Kasai T., Miyauchi K., Yamagishi S.I., Takeuchi M., Hiro T., Kimura T., Nakagawa Y. (2013). Relationship between Advanced Glycation End Products and Plaque Progression in Patients with Acute Coronary Syndrome: The JAPAN-ACS Sub-study. Cardiovasc. Diabetol..

[127] Naitoh T., Kitahara M., Tsuruzoe N. (2001). Tumor necrosis factor-α is induced through phorbol ester- and glycated human albumin-dependent pathway in THP-1 cells. Cell. Signal..

[128] Miele C., Riboulet A., Maitan M.A., Oriente F., Romano C., Formisano P., Giudicelli J., Beguinot F., Van Obberghen E. (2003). Human Glycated Albumin Affects Glucose Metabolism in L6 Skeletal Muscle Cells by Impairing Insulin-induced Insulin Receptor Substrate (IRS) Signaling through a Protein Kinase Cα-mediated Mechanism. J. Biol. Chem..

[129] Boyd A.C., Abdel-Wahab Y.H., McKillop A.M., McNulty H., Barnett C.R., O’Harte F.P.M., Flatt P.R. (2000). Impaired ability of glycated insulin to regulate plasma glucose and stimulate glucose transport and metabolism in mouse abdominal muscle. Biochim. Biophys. Acta Gen. Subj..

[130] Hunter S.J., Boyd A.C., O’Harte F.P., McKillop A.M., Wiggam M.I., Mooney M.H., McCluskey J.T., Lindsay J.R., Ennis C.N., Gamble R. (2003). Demonstration of glycated insulin in human diabetic plasma and decreased biological activity assessed by euglycemic-hyperinsulinemic clamp technique in humans. Diabetes.

[131] Cai W., Ramdas M., Zhu L., Chen X., Striker G.E., Vlassara H. (2012). Oral advanced glycation endproducts (AGEs) promote insulin resistance and diabetes by depleting the antioxidant defenses AGE receptor-1 and sirtuin 1. Proc. Natl. Acad. Sci. USA.

[132] Zhou Y.J., Wang J.H., Zhang J. (2006). Hepatocyte growth factor protects human endothelial cells against advanced glycation end products-induced apoposis. Biochem. Biophys. Res. Commun..

[133] Li Y., Li J., Cui L., Lai Y., Yao Y., Zhang Y., Pang X., Wang J., Liu X. (2013). Inhibitory effect of atorvastatin on AGE-induced HCAEC apoptosis by upregulating HSF-1 protein. Int. J. Biol. Macromol..

[134] Inagaki Y., Yamagishi S., Okamoto T., Takeuchi M., Amano S. (2003). Pigment epithelium-derived factor prevents advanced glycation end products-induced monocyte chemoattractant protein-1 production in microvascular endothelial cells by suppressing intracellular reactive oxygen species generation. Diabetologia.

[135] Ishibashi Y., Matsui T., Takeuchi M., Yamagishi S.I. (2010). Glucagon-like peptide-1 (GLP-1) inhibits advanced glycation end product (AGE)-induced up-regulation of VCAM-1 mRNA levels in endothelial cells by suppressing AGE receptor (RAGE) expression. Biochem. Biophys. Res. Commun..

[136] Yamagishi S., Fujimori H., Yonekura H., Yamamoto Y., Yamamoto H. (1998). Advanced glycation endproducts inhibit prostacyclin production and induce plasminogen activator inhibitor-1 in human microvascular endothelial cells. Diabetologia.

[137] Madamanchi N.R., Vendrov A., Runge M.S. (2005). Oxidative stress and vascular disease. Arterioscler. Thromb. Vasc. Biol..

[138] Barbato J.E., Tzeng E. (2004). Nitric oxide and arterial disease. J. Vasc. Surg..

[139] Yamagishi S.I., Inagaki Y., Okamoto T., Amano S., Koga K., Takeuchi M., Makita Z. (2002). Advanced glycation end product-induced apoptosis and overexpression of vascular endothelial growth factor and monocyte chemoattractant protein-1 in human-cultured mesangial cells. J. Biol. Chem..

[140] Antonetti D. (2009). Eye vessels saved by rescuing their pericyte partners. Nat. Med..

[141] Moore T.C., Moore J.E., Kaji Y., Frizzell N., Usui T., Poulaki V., Campbell I.L., Stitt A.W., Gardiner T.A., Archer D.B. (2003). The role of advanced glycation end products in retinal microvascular leukostasis. Investig. Ophthalmol. Vis. Sci..

[142] Schulz J.B., Lindenau J., Seyfried J., Dichgans J. (2000). Glutatione, oxidative stress and neurodegeneration. Eur. J. Biochem..

[143] Calabrese V., Cornelius C., Mancuso C., Lentile R., Stella A.G., Butterfield D.A., Uppu R., Murthy S., Pryor W., Parinandi N. (2010). Redox Homeostasis and Cellular Stress Response in Aging and Neurodegeneration. Free Radicals and Antioxidant Protocols. Methods in Molecular Biology.

[144] Vicente Miranda H., Outeiro T.F. (2010). The sour side of neurodegenerative disorders: The effects of protein glycation. J. Pathol..

[145] Mastrocola R. (2017). AGEs and neurodegeneration: The Nrf2/glyoxalase-1 interaction. Oncotarget.

[146] Srikanth V., Maczurek A., Phan T., Steele M., Westcott B., Juskiw D., Münch G. (2011). Advanced glycation endproducts and their receptor RAGE in Alzheimer’s disease. Neurobiol. Aging.

[147] Kaufmann E., Boehm B.O., Süssmuth S.D., Kientsch-Engel R., Sperfeld A., Ludolph A.C., Tumani H. (2004). The advanced glycation end-product N ε-(carboxymethyl)lysine level is elevated in cerebrospinal fluid of patients with amyotrophic lateral sclerosis. Neurosci. Lett..

[148] Sasaki N., Takeuchi M., Chowei H., Kikuchi S., Hayashi Y., Nakano N., Ikeda H., Yamagishi S.-I., Kitamoto T., Saito T. (2002). Advanced glycation end products (AGE) and their receptor (RAGE) in the brain of patients with Creutzfeldt-Jakob disease with prion plaques. Neurosci. Lett..

[149] Ko S., Ko H., Chu K.H., Shieh T., Chi T., Chen H., Chang W., Chang S. (2015). The Possible Mechanism of Advanced Glycation End Products (AGEs) for Alzheimer’s Disease. PLoS ONE.

[150] Li J.J., Voisin D., Quiquerez A.L., Bouras C. (1994). Differential expression of advanced glycosylation end-products in neurons of different species. Brain Res..

[151] Münch G., Mayer S., Michaelis J., Hipkiss A.R., Riederer P., Müller R., Neumann A., Schinzel R., Cunningham A.M. (1997). Influence of advanced glycation end-products and AGE-inhibitors on nucleation-dependent polymerization of β-amyloid peptide. Biochim. Biophys. Acta Mol. Basis Dis..

[152] Li Y.M., Dickson D.W. (1997). Enhanced binding of advanced glycation endproducts (AGE) by ApoE4 isoform links the mechanisms of plaque deposition in Alzheimer’s disease. Neurosci. Lett..

[153] Ledesma M.D., Bonay P., Avila J. (1995). τ Protein from Alzheimer’s Disease Patients Is Glycated at Its Tubulin-Binding Domain. J. Neurochem..

[154] Li X.H., Lv B.L., Xie J.Z., Liu J., Zhou X.W., Wang J.Z. (2012). AGEs induce Alzheimer-like tau pathology and memory deficit via RAGE-mediated GSK-3 activation. Neurobiol. Aging.

[155] Kalia L.V., Lang A.E. (2015). Parkinson’s disease. Lancet.

[156] Forno L.S. (1996). Neuropathology of Parkinson’s disease. J. Neuropathol. Exp. Neurol..

[157] Münch G., Lüth H., Wong A., Arendt T., Hirsch E., Ravid R., Riederer P. (2000). Crosslinking of alpha-synuclein by advanced glycation endproducts—An early pathophysiological step in Lewy body formation?. J. Chem. Neuroanat..

[158] Lee D., Park C.W., Paik S.R., Choi K.Y. (2009). The modification of α-synuclein by dicarbonyl compounds inhibits its fibril-forming process. Biochim. Biophys. Acta Proteins Proteom..

[159] Padmaraju V., Bhaskar J.J., Prasada Rao U.J., Salimath P.V., Rao K.S. (2011). Role of advanced glycation on aggregation and DNA binding properties of α-synuclein. J. Alzheimer’s Dis..

[160] Chen L., Wei Y., Wang X., He R. (2010). Ribosylation rapidly induces α-synuclein to form highly cytotoxic molten globules of advanced glycation end products. PLoS ONE.

[161] Van Heijst J.W., Niessen H.W., Hoekman K., Schalkwijk C.G. (2005). Advanced glycation end products in human cancer tissues: Detection of Nepsilon-(carboxymethyl)lysine and argpyrimidine. Ann. N. Y. Acad. Sci..

[162] Foster D., Spruill L., Walter K.R., Nogueira L.M., Fedarovich H., Turner R.Y., Ahmed M., Salley J.D., Ford M.E., Findlay V.J. (2014). AGE metabolites: A biomarker linked to cancer disparity?. Cancer Epidemiol. Biomark. Prev..

[163] Turner D.P. (2017). The Role of Advanced Glycation End-Products in Cancer Disparity.

[164] Turner D.P. (2015). Advanced glycation end-products: A biological consequence of lifestyle contributing to cancer disparity. Cancer Res..

[165] Yang S., Pinney S.M., Mallick P., Ho S.M., Bracken B., Wu T. (2015). Impact of Oxidative Stress Biomarkers and Carboxymethyllysine (an Advanced Glycation End Product) on Prostate Cancer: A Prospective Study. Clin. Genitourin. Cancer.

[166] Rodriguez-Teja M., Gronau J.H., Breit C., Zhang Y.Z., Minamidate A., Caley M.P., McCarthy A., Cox T.R., Erler J.T., Gaughan L. (2015). AGE-modified basement membrane cooperates with Endo180 to promote epithelial cell invasiveness and decrease prostate cancer survival. J. Pathol..

[167] Sharaf H., Matou-Nasri S., Wang Q., Rabhan Z., Al-Eidi H., Al Abdulrahman A., Ahmed N. (2015). Advanced glycation endproducts increase proliferation, migration and invasion of the breast cancer cell line MDA-MB-231. Biochim. Biophys. Acta Mol. Basis Dis..

[168] Riehl A., Németh J., Angel P., Hess J. (2009). The receptor RAGE: Bridging inflammation and cancer. Cell Commun. Signal..

[169] Rojas A., González I., Morales E., Pérez-Castro R., Romero J., Figueroa H. (2011). Diabetes and cancer: Looking at the multiligand/RAGE axis. World J. Diabetes.

[170] Elangovan I., Thirugnanam S., Chen A., Zheng G., Bosland M.C., Kajdacsy-Balla A., Gnanasekar M. (2012). Targeting receptor for advanced glycation end products (RAGE) expression induces apoptosis and inhibits prostate tumor growth. Biochem. Biophys. Res. Commun..

[171] Ishibashi Y., Matsui T., Takeuchi M., Yamagishi S. (2013). Metformin inhibits advanced glycation end products (AGEs)-induced growth and VEGF expression in MCF-7 breast cancer cells by suppressing AGEs receptor expression via AMP-activated protein kinase. Horm. Metab. Res..

[172] Pastino A.K., Greco T.M., Mathias R.A., Cristea I.M., Schwarzbauer J.E. (2017). Stimulatory effects of advanced glycation endproducts (AGEs) on fibronectin matrix assembly. Matrix Biol..

[173] Aggarwal B.B., Sundaram C., Malani N., Ichikawa H. (2007). Curcumin: The Indian solid gold. Adv. Exp. Med. Biol..

[174] Sajithlal G.B., Chithra P., Chandrakasan G. (1998). Effect of curcumin on the advanced glycation and cross-linking of collagen in diabetic rats. Biochem. Pharmacol..

[175] Jain S.K., Rains J., Jones K. (2006). Effect of curcumin on protein glycosylation, lipid peroxidation, and oxygen radical generation in human red blood cells exposed to high glucose levels. Free Radic. Biol. Med..

[176] Ghoneim A.I., Abdel-Naim A.B., Khalifa A.E., El-Denshary E.S. (2002). Protective effects of curcumin against ischaemia/reperfusion insult in rat forebrain. Pharmacol. Res..

[177] Kowluru R.A., Kanwar M. (2007). Effects of curcumin on retinal oxidative stress and inflammation in diabetes. Nutr. Metab..

[178] Nishiyama T., Mae T., Kishida H., Tsukagawa M., Mimaki Y., Kuroda M., Sashida Y., Takahashi K., Kawada T., Nakagawa K. (2005). Curcuminoids and sesquiterpenoids in turmeric (*Curcuma longa* L.) Suppress an increase in blood glucose level in type 2 diabetic KK-Aγ mice. J. Agric. Food Chem..

[179] Sharma S., Kulkarni S.K., Chopra K. (2006). Curcumin, the active principle of turmeric (*Curcuma longa*), ameliorates diabetic nephropathy in rats. Clin. Exp. Pharmacol. Physiol..

[180] Chen Q., Wang T., Li J., Wang S., Qiu F., Yu H., Zhang Y., Wang T. (2017). Effects of natural products on fructose-induced nonalcoholic fatty liver disease (NAFLD). Nutrients.

[181] Maithilikarpagaselvi N., Sridhar M.G., Swaminathan R.P., Sripradha R., Badhe B. (2016). Curcumin inhibits hyperlipidemia and hepatic fat accumulation in high-fructose-fed male Wistar rats. Pharm. Biol..

[182] Babu P.V.A., Sabitha K.E., Shyamaladevi C.S. (2008). Effect of green tea extract on advanced glycation and cross-linking of tail tendon collagen in streptozotocin induced diabetic rats. Food Chem. Toxicol..

[183] Dearlove R.P., Greenspan P., Hartle D.K., Swanson R.B., Hargrove J.L. (2008). Inhibition of Protein Glycation by Extracts of Culinary Herbs and Spices. J. Med. Food.

[184] Frei B., Higdon J.V. (2003). Antioxidant activity of tea polyphenols in vivo: Evidence from animal studies. J. Nutr..

[185] Kashima M. (1999). Effects of catechins on superoxide and hydroxyl radical. Chem. Pharm. Bull..

[186] Rizvi S.I., Zaid M.A., Anis R., Mishra N. (2005). Protective role of tea catechins against oxidation-induced damage of type 2 diabetic erythrocytes. Clin. Exp. Pharmacol. Physiol..

[187] Li X., Zheng T., Sang S., Lv L. (2014). Quercetin Inhibits Advanced Glycation End Product Formation by Trapping Methylglyoxal and Glyoxal. J. Agric. Food Chem..

[188] Bors W., Heller W., Michel C., Saran M. (1990). Flavonoids as antioxidants: Determination of radical-scavenging efficiencies. Methods Enzymol..

[189] Yokozawa T., Kashiwada Y., Hattori M., Chung H.Y. (2002). Study on the Components of Luobuma with Peroxynitrite-Scavenging Activity. Biol. Pharm. Bull. Pharm. Bull..

[190] Yokozawa T., Nakagawa T. (2004). Inhibitory effects of Luobuma tea and its components against glucose-mediated protein damage. Food Chem. Toxicol..

[191] Ahmad M.S., Pischetsrieder M., Ahmed N. (2007). Aged garlic extract and S-allyl cysteine prevent formation of advanced glycation endproducts. Eur. J. Pharmacol..

[192] Venkateswara Rao P., Kiran S., Rohini P., Bhagyasree P. (2017). Flavonoid: A review on Naringenin. J. Pharmacogn. Phytochem..

[193] Teng J., Li Y., Yu W., Zhao Y., Hu X., Tao N., Wang M. (2018). Naringenin, a common flavanone, inhibits the formation of AGEs in bread and attenuates AGEs-induced oxidative stress and inflammation in RAW264.7 cells. Food Chem..

[194] Chen Y.J., Kong L., Tang Z.Z., Zhang Y.M., Liu Y., Wang T.Y., Liu Y.W. (2019). Hesperetin ameliorates diabetic nephropathy in rats by activating Nrf2/ARE/glyoxalase 1 pathway. Biomed. Pharmacother..

[195] Zhou Q., Cheng K.W., Gong J., Li E.T.S., Wang M. (2019). Apigenin and its methylglyoxal-adduct inhibit advanced glycation end products-induced oxidative stress and inflammation in endothelial cells. Biochem. Pharmacol..

[196] Zhao Y., Wang P., Sang S. (2019). Dietary Genistein Inhibits Methylglyoxal-Induced Advanced Glycation End Product Formation in Mice Fed a High-Fat Diet. J. Nutr..

[197] Navarro M., Morales F.J., Ramos S. (2017). Olive leaf extract concentrated in hydroxytyrosol attenuates protein carbonylation and the formation of advanced glycation end products in a hepatic cell line (HepG2). Food Funct..

[198] Carrizzo A., Forte M., Damato A., Trimarco V., Salzano F., Bartolo M., Maciag A., Puca A.A., Vecchione C. (2013). Antioxidant effects of resveratrol in cardiovascular, cerebral and metabolic diseases. Food Chem. Toxicol..

[199] Abbasi Oshaghi E., Goodarzi M.T., Higgins V., Adeli K. (2017). Role of resveratrol in the management of insulin resistance and related conditions: Mechanism of action. Crit. Rev. Clin. Lab. Sci..

[200] Kulkarni S.S., Cantó C. (2015). The molecular targets of resveratrol. Biochim. Biophys. Acta Mol. Basis Dis..

[201] Yılmaz Z., Kalaz E.B., Aydın A.F., Olgaç V., Doğru-Abbasoğlu S., Uysal M., Koçak-Toker N. (2018). The effect of resveratrol on glycation and oxidation products in plasma and liver of chronic methylglyoxal-treated rats. Pharmacol. Rep..

[202] Moridi H., Karimi J., Sheikh N., Goodarzi M.T., Saidijam M., Yadegarazari R., Khazaei M., Khodadadi I., Tavilani H., Piri H. (2015). Resveratrol-dependent down-regulation of receptor for advanced glycation end-products and oxidative stress in kidney of rats with diabetes. Int. J. Endocrinol. Metab..

[203] Khazaei M., Karimi J., Sheikh N., Goodarzi M.T., Saidijam M., Khodadadi I., Moridi H. (2016). Effects of Resveratrol on Receptor for Advanced Glycation End Products (RAGE) Expression and Oxidative Stress in the Liver of Rats with Type 2 Diabetes. Phyther. Res..

[204] Schröter D., Höhn A. (2018). Role of Advanced Glycation End Products in Carcinogenesis and Their Therapeutic Implications. Curr. Pharm. Des..

